# Cerebellar mutism syndrome and its relation to cerebellar cognitive and affective function: Review of the literature

**DOI:** 10.4103/0972-2327.61272

**Published:** 2010

**Authors:** Ozlem Yildiz, Serdar Kabatas, Cem Yilmaz, Nur Altinors, Belma Agaoglu

**Affiliations:** Department of Child and Adolescent Psychiatry, Kocaeli University, Faculty of Medicine, Kocaeli, Turkey; 1 Department of Neurosurgery, Baskent University, Ankara, Turkey

**Keywords:** Cerebellar cognitive function, cerebellar mutism syndrome, neuropsychology

## Abstract

Tumors of the cerebellum and brainstem account for half of all brain tumors in children. The realization that cerebellar lesions produce clinically relevant intellectual disability makes it important to determine whether neuropsychological abnormalities occur in long-term survivors of pediatric cerebellar tumors. Little is known about the neurobehavioral sequale resulting specifically from the resection of these tumors in this population. We therefore reviewed neuropsychological findings associated with postoperative cerebellar mutism syndrome and discuss the further implications for cerebellar cognitive function.

## Introduction

Postoperative cerebellar mutism syndrome (CMS) is characterized by severely diminished or absent speech output as well as other neurological, cognitive, and behavioral impairments. The incidence of CMS in children who underwent surgery of the cerebellum is estimated to be between 8 and 31%.[1,2] CMS has been reported in over 200 cases to date.[3,4] Theories about the pathophysiology of CMS have evolved along with our understanding of the cerebellum as an important structure in the distributive neurocircuitry underlying complex speech, cognition, and behavior.[5] In addition to mutism, the most commonly observed findings are ataxia, hypotonia, and emotional lability. Personality and emotional disturbances includes irritability, disinhibition, inattention, and lability of affect with poor cognitive and behavioral modulation.[6,7]

## Cerebellar Mutism Syndrome and Neurocognitive Features

Recent research studies suggest that neurological and cognitive impairments in CMS often persist. A prospective study evaluated the neurological status of patients 1 year post-diagnosis based on the presence and severity of ataxia, language difficulties, and other cognitive deficits.[7] Of the 46 patients who had postoperative CMS initially rated severe, residual deficits were common, including 92% with ataxia, 66% with speech and language dysfunction, and 59% with global intellectual impairment. Of the 52 patients with moderate CMS, 78% had ataxia, 25% had speech and language dysfunction, and 17% had global intellectual impairment. Thus, impairment in these domains was common and was also directly related to the severity of CMS. Riva and Giorgi have shown neuropsychological problems a few weeks after cerebellar tumor resection, and prior to further treatment such as radiotherapy or chemotheraphy.[8] Their results reveal a localization related pattern, with problems of auditory sequential memory and language processing after right-sided cerebellar tumor and deficits in spatial and visual memory after left-sided tumor. Lesions to the vermis led to post-surgical mutism, which evolved into speech and language disorders as well as behavioral disturbances ranging from irritability to those reminiscent of mutism.[8] Levisohn and colleagues presented a retrospective study of neuropsychological problems of children during the first 2 years after resection of a cerebellar tumor.[6] These children had problems comparable with cognitive affective syndrome in adults with dysfunction in visual-spatial tasks, language sequencing, memory, and regulation of affect. There was no localization related pattern as in the patients of Riva and Giorgi.[6,8] Karatekin *et al*. studied the effect of isolated cerebellar hemispheric tumors post-operatively and compared them with the effect of temporal tumors.[9] After cerebellar lesions, children had a neuropsychological pattern characterized by executive function problems, which was different to those who had suffered temporal tumors.[9] In a unique case study of CMS, Ozgur *et al*. described a 5 year old with medulloblastoma and associated hydrocephalus.[10] One day post-surgical resection, the patient exhibited cerebellar dysmetria, dysdiadokinesia, and mutism. Although motor symptoms continued to improve over the next several weeks, the mutism remained. Serendipitously, the patient was exposed to familiar and favorite music and began singing without prompts, but remained mute without the music. However, the patient's speech recovered quickly thereafter. Other symptomatology during that period included decreased initiation, poor regulatory control and attention, impaired language comprehension, and emotional apathy and irritability [Table 1].[6,8,10–12] Furthermore, transient postoperative cerebellar mutism was reported as an extreme form of cerebellar dysarthria due to surgical evacuation of a spontaneous vermian hematoma in an 8-year-old boy.[13] Additionally, in a unique case study, two patients -one child and one adult- who developed mutism, oropharyngeal apraxia, and dysarthria after cerebellar surgery were reported as complications due to possible involvement of vermian and paravermian structures.[14] Thus, some answers may be found through a better understanding of the injured areas that underlie each of the key responses of formation of the CMS.

**Table 1:** Review of cerebellar tumor types and their effects for neurocognitive dysfunction

Authors	Number of cases	Age intervals (years)	Cerebellar tumor types (Number of cases)	Neurocognitive impairments
Riva and Giorgi[25]	26	6–12	Medulloblastoma (11); astrocytoma (15)	*Medulloblastoma group*: (6 of the 11 exhibited CMS), language deficits, executive dysfunction (poor set-shifting, verbal initiation), behavioral disturbance.*Astrocytoma group*: global receptive and expressive language deficits, executive dysfunction (verbal initiation, planning, set-shifting), deficit in processing speed
Levisohn *et al*.[18]	19	3–14	Medulloblastoma (11); astrocytoma (7); ependymoma (1)	Expressive language deficits, word-finding difficulties, visual-spatial functions, visual- spatial memory and affective impairments, executive dysfunction
Aarsen *et al*.[1]	23	?	Astrocytoma	*Right cerebellar hemisphere resection*: language and verbal memory impairments *Left cerebellar hemisphere resection*: visuo-spatial and nonverbal memory impairments
Ronning *et al*.[27]	23	6–9	Medullolastoma treatedwith radio-chemotherapy (11); astrocytoma (12)	Medulloblastoma group performed poorer than the astrocytoma group on intelligence, motor function, attention deficit, psychomotor speed, verbal and visual memory
Ozgur *et al*.[22]	1	5	Medulloblastoma and associated hydrocephalus	Decreased verbal initiation, poor regulatory control, attention impairment, language comprehension, emotional apathy, irritability

The behavioral changes observed after posterior fossa surgery have often been interpreted as symptoms of reactive depression. Pollack and colleagues descibed personality changes and emotional lability.[1] In another study, their findings supported an association between extensive vermis damage and impaired regulation of affect, including irritability, impulsivity, disinhibition, and lability of affect with poor attentional and behavioral modulation.[6] This pattern is consistent with other clinical evidence of a relationship between vermis abnormalities and affective disturbance, such as those seen in children with vermal agenesis, in adults with cerebellar cognitive affective syndrome, and in the posterior fossa syndrome that develops in 15% of children who undergo midline cerebellar surgery and that is characterized by transient postoperative mutism as well as inconsolable whining, emotional lability, withdrawal, and apathy.[1,15]

The role of the cerebellum in emotional behavior has been demonstrated in primates with cerebellar lesions. Other evidence that the cerebellum plays a role in higher order behaviors comes from imaging studies of children with neuropsychiatric and genetic disorders such as attention deficit/hyperactivity disorder (ADHD), autism, developmental dyslexia, Fragile X, Down's syndrome, and schizophrenia.[16–23] Regarding further detailed evaluation of cerebellar function, positron emission tomography (PET) and functional magnetic resonance imaging (fMRI) studies have shown cerebellar activity in healthy control subjects in different cognitive tasks. Independent of motor involvement, different areas of the cerebellum were activated by non-spatial shifting attention tasks or selective attention tasks in two studies.[24,25] Right cerebellar activation has been reported in verbal fluency paradigms. The site of activation (right or left cerebellar hemisphere) seems to be contralateral to the activation of the frontal cortex, even under conditions of different language dominance. Greater cognitive demands in verbal fluency tasks seem to lead to more extensive cerebellar activation.[26] Furthermore, recent functional imaging data point at a contribution of the right cerebellar hemisphere, concomitant with language dominant dorsolateral and medial frontal areas, to the temporal organization of a prearticulatory verbal code (“inner speech”), in terms of the sequencing of syllable strings at a speaker 's habitual speech rate. Besides motor control, this network also appears to be engaged in executive functions, e.g. subvocal rehearsal mechanisms of verbal working memory and seems to be recruited during distinct speech perception tasks. Taken together, thus, a prearticulatory verbal code bound to reciprocal right cerebellar/ left frontal interactions might represent a common platform for a variety of cerebellar engagements in cognitive functions.[27] In addition to this, Levisohn *et al*. found that patients with CMS including affective changes also demonstrated cognitive impairment, but patients with cognitive changes did not necessarily show CMS and affect disturbance.[6] This finding is consistent with the hypothesis that affect regulation is principally a function of the vermis and fastigial nucleus, but both the vermis and the cerebellar hemispheres are involved in executive, linguistic, and visual-spatial functions.[28,29] Several other studies have demonstrated similar late effects in patients with CMS, including diminished processing speed, poor verbal initiation and other language deficits, impaired attention, and executive functions (e.g., setshifting, novel problem solving), as well as memory deficits.[11,30] Additionally, memory deficits might be seen in isolated cerebellar lesions. As previously stated in the literature by case reports[31–33] or series,[34–37] the high incidence of memory deficits in patients with cerebellar lesions proves that the cerebellum functions in higher cognition tasks.

## Pharmacological Enhancement of Cognitive and Behavioral Deficits

In the recent literature, most studies have encountered and focused on pharmacological therapy for cognitive and behavioral deficits due to traumatic brain injuries in adults. Several cases reported that treatment with drugs (e.g., bromocriptine) showed a relationship with cerebellar mutism and cognitive dysfunction in children.[38–40] Bromocriptine, a direct dopamine agonist affecting primarily D2 receptors, also seems to have activity with respect to specific executive function and attentional abilities in both animal and human studies. Rats subjected to a controlled cortical impact show improved working memory and spatial learning abilities, but not motor abilities, when treated chronically with bromocriptine.[41] The studies with normal humans using single low (2.5 mg) doses have yielded complex results, with some subjects benefiting and some deteriorating, depending on their baseline working memory capacities and the timing of drug administration.[42] Hence, during posterior fossa surgery, monoaminergic cell groups and particularly dopaminergic cell groups may be damaged, resulting in akinetic mutism. The dopamine agonist bromocriptine may be a useful therapeutic agent for this complication.

Attentional problems and disinhibited behavior are common sequelae of children with posterior fossa surgery. The success of methylphenidate in children with attention and behavior disorders has prompted researchers and clinicians to use the drug for similar disorders seen in acquired brain injuries. Siddall provided a review of the use of methylphenidate in traumatic brain injury based on ten published studies on the subject. The author concluded that methylphenidate is likely to improve memory, attention, concentration, and mental processing, but that its effects on behavior have not been determined.[43] Optimal dosing, timing and length of treatment, and long- term effects were considered to need further double-blinded placebo-controlled studies. In a randomized placebo-controlled double-blinded study, Kim and Kim studied the effect of a single dose (20 mg) of methylphenidate on working memory and visual-spatial attention in 18 patients with traumatic brain injury.[44] The severity of the injury was not stated, but all subjects were said to be symptomatic and in a chronic phase. According to the authors, methylphenidate significantly improved response accuracy in both these functions and shortened reaction time in the working memory task. Their clinical message that methylphenidate appears to have the most effect on the working memory is an oversimplification when one considers that only two cognitive tests were used.[44]

Atomoxetine is a selective norepinephrine uptake inhibitor which has mainly been used in the treatment of ADHD. Recent studies have shown that it may enhance executive function, decrease fatigue, and enhance short-term plasticity.[45,46] Furthermore, it may be a useful therapeutic agent due to the therapy of inattention and executive dysfunction after posterior fossa surgery. However, there are few studies to demonstrate its real effects.

In the recent studies, other drugs that have been used in the treatment of cognitive and affective disorders include cholinergic agents (donepezil), dopaminergic agents (amantadine, selegiline), antidepressants (selective serotonin reuptake inhibitors) and antiepileptics (valproic acit, carbamazepine) [Table 2].[47–50] Further detailed studies are necessary to understand the effects of these drugs in children.

**Table 2:** Treatment modalities regarding the most common symptoms for the cerebellar mutism syndrome and its relation to cerebellar cognitive and affective function

Symptom	First-line alternatives	Other options
Memory problems	Methylphenidate, anticholinerjic agents, dopamine agonist	Atomoxetine, modafinil
Attentional problems	Methylphenidate, anticholinerjic agents, amantadine	Atomoxetine, modafinil
Executive dysfunction	Methylphenidate, anticholinerjic agents, amantadine	Atomoxetine, modafinil
Depression	Selective serotonin reuptake inhibitors	Newer antidepressants, methylphenidate
Emotional lability	Antiepileptic ajans (carbamazepine, valproate)	Lamotrigine, Atypical antipsychotics
Fatigue	Anticolinerjic ajans, methylphenidate	Atomoxetine, modafinil
Poor initiation, apathy	Selective serotonin reuptake inhibitors, Anticolinerjic ajans, amantadine	Methylphenidate, Atomoxetine, modafinil
Slowness of information processing	Methylphenidate, anticholinerjic agents	Atomoxetine, modafinil

In conclusion, these findings show that the cerebellum is important in cognitive and affective functioning. Besides the similarity of frontal impairment of executive functions, there are also additional challenges, especially in visual-spatial domains, attention and short-term memory. The pattern of these dysfunctions, involving many different cognitive functions, supports the idea that cerebellar cognitive and affective symptoms cannot be explained by one uniform cerebellar dysfunction, but are rather a consequence of the many pathways connecting the cerebellum and the cerebrum in both directions. It is important to point to the psychopathological problems of these children. Both early and long-term neuropsychological follow-up and specific intervention until integration into professional life seem mandatory for children after posterior fossa tumors. Follow-up of all patients treated for a posterior fossa tumor in childhood should include extensive neuropsychological testing at regular intervals. This may be of benefit for school planning and later work planning.

## Summary

In children with CMS, executive, lingual and affective dysfunctions are seem to be related to tumor type, localization, time frame of the diagnosis, and receiving radio-/ chemotherapy after the operation. As briefly, in patients with medulloblastoma, at postoperative radio-/ chemotherapy period, and in the early age of diagnose, the incidence of CMS with executive, lingual, and affective dysfunctions are common. In addition, in patients with vermian lesions affective dysfunction and in patients with hemispherical lesions lingual and executive dysfunctions are usually affected. The improving survivals of these patients with the advances in treatment modalities may decline their life quality if they were evaluated only by their neurological deficits. Prior detection of problems due to executive, language and affective dysfunction, and multidisiplinary management strategies should be considered in the follow-up therapies. Patients have improved quality of life if they receive pharmacological, speech threapy, and indivudual educational support with physiciatric examination. Furthermore, a detailed understanding of cerebellary functions may give further insight into ADHD, autism, and schizophrenia-like physiciatric disorders.
